# Preparation and Evaluation of Silymarin-Loaded Solid Eutectic for Enhanced Anti-Inflammatory, Hepatoprotective Effect: *In Vitro*–*In Vivo* Prospect

**DOI:** 10.1155/2021/1818538

**Published:** 2021-11-10

**Authors:** Abdulla Sherikar, Mohd Usman Mohd Siddique, Mahesh More, Sameer N. Goyal, Milan Milivojevic, Saad Alkahtani, Saud Alarifi, Md Saquib Hasnain, Amit Kumar Nayak

**Affiliations:** ^1^Department of Pharmacology Shri Vile Parle Kelavani Mandal's Institute of Pharmacy, Dhule, Maharashtra 424001, India; ^2^Department of Pharmaceutical Chemistry, Shri Vile Parle Kelavani Mandal's Institute of Pharmacy, Dhule, Maharashtra 424001, India; ^3^Department of Pharmaceutics, Dr. Rajendra Gode College of Pharmacy, Dist Buldhana (M.S.), 443 101, Malkapur, India; ^4^Department of Chemical Engineering, Faculty of Technology and Metallurgy, University of Belgrade, Belgrade 11000, Serbia; ^5^Department of Zoology, College of Science, King Saud University, P.O. Box 2455, Riyadh, Saudi Arabia; ^6^Department of Pharmacy, Palamau Institute of Pharmacy, Chianki, Daltonganj, 822102 Jharkhand, India; ^7^Department of Pharmaceutics, Seemanta Institute of Pharmaceutical Sciences, Mayurbhanj, 757086 Odisha, India

## Abstract

Solubility of phytochemicals is a major concern for drug delivery, permeability, and their biological response. However, advancements in the novel formulation technologies have been helping to overcome these challenges. The applications of these newer technologies are easy for commercialization and high therapeutic outcomes compared to conventional formulations. Considering these facts, the present study is aimed to prepare a silymarin-loaded eutectic mixture with three different ratios of Polyvinylpyrrolidone K30 (PVP K30) and evaluating their anti-inflammatory, and hepatoprotective effects. The preliminary phytochemical and characterization of silymarin, physical mixture, and solid dispersions suggested and successfully confirmed the formation of solid dispersion of silymarin with PVP K30. It was found that the solubility of silymarin was increased by 5-fold compared to pure silymarin. Moreover, the *in vitro* dissolution displayed that 83% of silymarin released within 2 h with 2.8-fold increase in dissolution rate compared to pure silymarin. Also, the *in vivo* study suggested that the formulation significantly reduced the carbon tetrachloride- (0.8620 ± 0.05034^∗∗^ for 1 : 3 ratio), paracetamol- (0.7300 ± 0.01517^∗∗^ for 1 : 3 ratio), and ethanol- (0.8100 ± 0.04037^∗∗^ for 1 : 3 ratio) induced hepatotoxicity in rats. Silymarin solid dispersion was prepared using homogenization methods that have prominent anti-inflammatory effect (0.6520 ± 0.008602^∗∗^ with 8.33%) in carrageenan-induced rat paw model.

## 1. Introduction

Solid solution is an interchangeable solution state while solute interacting strongly in the form of eutectics. Solid dispersion method maximizes interaction with water and profoundly incorporates hydrogen bonds. Furthermore, it allows the intercalation of the lipophilic substance centrally giving the odor of hydrophilic monolayer polymer. Solid dispersion is widely used and a well-explored technique for the enhancement of solubility at both laboratory and commercial scale [1]. But macerates of plants or animal displayed the limited solubility in aqueous environment, and recent literature showed that the organic extracts have better therapeutic potential as compared to the aqueous extracts. The possibility behind this is it may be due to the hydrophobic nature of most of the active constituents. These formulation challenges, mainly low solubility and poor bioavailability, limit the scope and commercial availability of phytochemicals [2, 3]. The formulation development of large number of phytoconstituents is also problematic. The different observations were noted while developing the formulation of phytoconstituents, mainly incompatibility with solvents, precipitation, phase separation, aggregation, etc. This creates an opportunity for the researchers to come up with different strategies for the enhancement of characterization of phytoconstituent extracts. For enhancing the bioavailability of phytoconstituents, novel solubility enhancement techniques like solid dispersion, complexation, nanocrystals, ultrasonication, and eutectic mixtures provide an alternative approach [4–8]. Silymarin (SL) is the active constituent of *Silybum marianum* L. Gaertn. (Family Asteraceae) traditionally used in the treatment of the liver, and it is regarded as a highly potent hepatoprotective agent [6, 9]. The major constituent of silymarin is silibinin (A and B), and it is found to be about 70–80% responsible for major therapeutic activity. The other flavanolignan components like silydianin, silycrystin, and isosilybin (A and B) also showed 10%, 20%, and 0.5% useful pharmacological activity, respectively. SL is an important class of phytopharmaceuticals having wide therapeutic applications as anticancer, antiviral, antifibrotic, etc. [10–12]. Despite this, it showed limited therapeutic outcome because of its poor solubility and limited bioavailability after oral administration. Furthermore, it undergoes first pass effect providing only 20–40% of therapeutic benefit. Orally administered silybin has not been detected in plasma due to poor solubility characteristics and oral absorption. Oral administration and first pass effect lead to very less bioavailability (approximately 0.95%) [13]. Various formulations of SL have been tried to enhance the solubility and bioavailability, but the significant results are still lacking. Overall, these factor forms the basis of present study, i.e., formulation of eutectic solid of silymarin. Many active pharmaceutical ingredients, including phytochemicals, have poor solubility and bioavailability. A group of researchers is actively working on the solubility enhancement techniques, including a new mechanism of solute–solvent or solute–solute interaction. In order to enhance the dissolution pattern of poorly soluble drug, the solid dispersion technique is widely employed [14, 15]. Moreover, this system is associated with formation of polymer conjugation of poorly soluble drugs through amorphization of drug in the presence of polymeric carrier [16, 17]. Polyvinylpyrrolidone K30(PVP K30), a derivatized longer chain, water-soluble, hydrophilic polymer, was used for making silymarin eutectic. Due to its nonirritant, nontoxic, biocompatible, and biodegradable characteristics of PVP K30, it is widely employed in drug delivery carrier. Furthermore, PVP K30 is a linear nonionic polymer which has wide range of pharmaceutical applications like solubility enhancement, protection of crystalline drug during processing, and delivery of drug [18, 19]. Also, Polyvinylpyrrolidone (PVP) not only forms complexes with the drug molecules, but also exhibits strong solute-solute interaction like hydrogen bonding, Van der Waals weak attraction, or London forces. For preparation of solid dispersion, the PVP is considered as a good candidate for enhancing solubility of drug that imparts protection to drug from loss due to external environment [19, 20]. The present study was aimed at enhancing the solubility and thereby bioavailability of SL by solid dispersion technique. The solid dispersion was prepared using appropriate proportions of PVP K30 and SL to be processed at optimum temperature. The silymarin solid dispersion (SDD) was conjugated with hydroxypropyl methylcellulose (HPMC) carrierto modulate long-term release. In the presence of PVP K30, solubility and dissolution characteristics of SL were set to be increased. *In vivo* pharmacokinetic study suggested that prepared silymarin solid dispersion has proven to have antioxidant and anti-inflammatory activity and hepatoprotective effect.

## 2. Experimental

### 2.1. Materials

Silymarin (SL) was received as a gift sample from BioXpert Innovations Pvt. Ltd., India. Polyvinylpyrrolidone K30 (PVP K30) and hydroxypropyl methylcellulose (HPMC) were made available from Loba Chemie Ltd., Mumbai, India. The remaining chemicals belong to analytical grade and are used as received unless it is specified.

### 2.2. Methods

#### 2.2.1. Preparation of Silymarin-Encapsulated Solid Dispersion

The phytochemical has limited solubility and thereby, limits the therapeutic response. For enhancement of solubility, phytoconstituents are mixed with either surfactant or polymeric blend [21]. Variable ratios of SL and PVP K30 were taken for improving the solubility. In order to prepare the SL-based solid dispersion (SSD), the solvent evaporation technique was employed. Different concentrations of PVP K30 were used along with SL to verify the solubility characteristics. Methanol was used as common volatile solvent for the preparation containing SL and PVP K30. The concentration ratios (1 : 1, 1 : 2, and 1 : 3) were taken with respect to SL and PVP K30. For further experimentation, concentration ratios of SL to PVP K30 (1 : 1, 1 : 2, and 1 : 3) were used to notify as silymarin solid dispersion (SSD1, SSD2, and SSD3), respectively. PVP K30 and SL were dissolved in methanol (15 mL). The solution was ultrasonicated for 15 min to remove traces of aggregates. The methanolic solution containing PVP K30 and SL was added dropwise in 50 mL water containing PEG 4000 (0.2%). The whole mixture was homogenized (IKA Homogenizer T25) for 40 min at 10000 rpm. The methanol was evaporated in the Rota evaporator at a reduced temperature. The remaining aqueous solution was lyophilized at -40°C, and dry powder was collected for analysis.

### 2.3. Characterization

Preliminary characterization of SL, PVP K30, solid dispersion, and physical mixture was done using sophisticated analytical instruments. The preliminary identification of SL was done using a UV-visible double-beam spectrophotometer (JASCO V630) and a Fourier transform infrared (FTIR) spectrophotometer with diffused reflectance assembly (JASCO 4100S) [22]. The stock solution containing SL was dissolved into 0.1 N HCl. The UV-visible spectra were recorded by scanning 200–400 nm. The maximum absorbance (*λ*_max_) from the spectrum was identified and used in further evaluations like drug content or drug release. The preliminary identification of SL, PVP K30, physical mixture (SL+PVP K30), and SSD was evaluated using FTIR spectroscopy. The individual solid component was mixed with predried KBr in 1 : 100 ratio (solid : KBr) and analyzed using an FTIR spectrophotometer. The vibrational intensities of obtained spectra were compared with standards. Solubility analysis of pure SL and solid dispersion was performed using the shake flask method, and concentration was estimated after 48 h period of time using UV-visible spectrophotometric absorbance. The absorbance was correlated with calibration curve plotted in the respective solution state with linearity (*R*^2^) 0.998.

### 2.4. *In Vivo* Pharmacological Study

#### 2.4.1. Animals

The male albino Wistar rats weighing 180-200 g used were procured from the animal house, Laxmi Biofarm, Alephata, Pune. The different grouping of rats was done and kept in polyacrylic cages (38 cm × 23 cm × 10 cm) and maintained under standard laboratory conditions (temperature (25 ± 3)°C) with dark and light cycle (12/12 h). The rats were allowed free access to a standard pellet diet and reversed osmosis water *ad libitum*. Before the initiation of the experiment, the period of one week was followed for the acclimatization of selected rats. *In vivo* pharmacological screening was conducted for pharmacological activity.

#### 2.4.2. *In Vivo* Anti-Inflammatory Activity (Carrageenan-Induced Paw Edema in Rats)

To induce acute inflammation in the paw of rats, 1% suspension of carrageenan in normal saline was prepared, and 0.1 mL subplantar injection was given to the right hind paw of rats. The digital plethysmometer (Orchid Scientific, Nashik) was used to measure paw volume to indicate acute inflammation at different time intervals like 0, 30, 60, 90, 120, and 180 min after carrageenan injection. The average foot swellings among the test and standard groups were regarded as a function of edema. The variations among the two readings were considered as the volume of edema. Moreover, the % inhibition of paw edema as a marker of anti-inflammatory activity by SSD was calculated by using.

(1) $$\begin{array}{c} \%\text{Edema}=\frac{C_{0}-C_{r}}{C_{0}}\times 100, \end{array}$$

where *C*_r_ is the average paw volume of the treated group and *C*_0_ is the average paw volume of the control group. The animals were grouped as follows: Group 1: control group, Group 2: standard drug treated (containing 1.16% diclofenac sodium) [23], Group 3: administered with SSD (1 : 1) 100 mg/kg, p.o., Group 4: administered with SSD (1 : 2) 100 mg/kg, p.o., and Group 5: administered with SSD (1 : 3) 100 mg/kg, p.o.

#### 2.4.3. *In Vivo* Hepatoprotective Activity

For hepatoprotective activity, 30 healthy albino Wistar rats of either sex were divided into 6 groups. Hepatoprotective activity was comparatively assessed using three different methods, such as carbon tetrachloride (CCl_4_) method, ethanol method, and paracetamol method. During study, each group consists of 6 rats, and dosing was done frequently as mentioned in the individual model.

*(1) Carbon Tetrachloride- (CCl_4_-) Induced Hepatotoxicity in Rats*. For the screening of *in vivo* hepatoprotective activity of SSD in CCl_4_-induced hepatotoxicity in rats, the animals were grouped as follows.

Group 1: the rats were administered with sodium carboxymethylcellulose (NaCMC 0.5%, p.o.) as a vehicle for six days.

Group 2: the rats were administered with vehicle (NaCMC 0.5%, p.o.) for six days, and on day 7, they were treated with CCl_4_, 1.5 mL/kg, p.o. [24].

Group 3: the rats were treated with standard drug, silymarin 100 mg/kg, p.o. [10], for six days, and on day 7, they were treated with CCl_4_, 1.5 mL/kg, p.o.

Groups 4, 5, and 6: the rats were treated with SSD (1 : 1, 1 : 2, and 1 : 3) 100 mg/kg, p.o., for six days, and on day 7, these were treated with CCl_4_, 1.5 mL/kg, p.o., respectively.

*(2) Ethanol-Induced Hepatotoxicity in Rats*. The animals were divided into different groups to screen in vivo hepatoprotective activity of SSD in ethanol-induced hepatotoxicity model.

Group 1: the rats were administered with 2% gum acacia (0.1 g/200 g b.w.) for 6 days.

Group 2: the rats were administered with vehicle and 2% gum acacia (0.1 g/200 g b.w.) for six days, and on day 7, they were treated with 3.76 g/kg b.w. ethanol (20%), p.o.

Group 3: the rats were treated with standard drug, silymarin 100 mg/kg b.w., p.o., respectively, for six days, and on day 7, they were treated with 3.76 g/kg b.w. ethanol (20%), p.o.

Groups 4, 5, and 6: the rats were treated with SSD (1 : 1, 1 : 2, and 1 : 3) 100 mg/kg, p.o., for six days, and on day 7, they were treated with 3.76 g/kg b.w. ethanol (20%), p.o., respectively [25].

*(3) Paracetamol-Induced Hepatotoxicity in Rats*. In the context of *in vivo* paracetamol-induced hepatotoxicity, the animals were divided into different groups.

Group 1: the rats were administered with 1% sodium-carboxymethylcellulose (NaCMC) 1 mL/kg b.w., p.o. for 6 days.

Group 2: the rats were administered with vehicle 1% NaCMC 1 mL/kg b.w., p.o., for six days, and on day 7, they were treated with 500 mg/kg b.w. paracetamol, p.o.

Group 3: the rats were treated with standard drug, silymarin 100 mg/kg b.w., p.o., respectively, for six days, and on day 7, they were treated with 500 mg/kg paracetamol, p.o.

Groups 4, 5, and 6: the rats were treated with SSD (1 : 1, 1 : 2, and 1 : 3) 100 mg/kg, p.o., for six days, and on day 7, they were treated with 500 mg/kg paracetamol, p.o., respectively [23].

Hepatoprotective activity was biochemically evaluated for each model, such as alanine aminotransferase (ALT), aspartate aminotransferase (AST), alkaline phosphate (ALP), and total bilirubin.

After 48 h of administration of each hepatotoxic agent into a separate group of animals, blood samples were collected from the retro-orbital plexus. The blood was transferred into previously labelled centrifuge tube and allowed to clot for 30 min at room temperature. The tubes were centrifuged at 2500 rpm for 10 min, and serum was separated. The separated serum was analyzed using standard biochemical kits for the estimation of ALT, AST, ALP, and total bilirubin.

### 2.5. Statistical Analysis

The data were presented as the mean ± SEM. The data were analyzed by GraphPad Prism (version 5.0) using one-way ANOVA, followed by Dunnett's test.

## 3. Results and Discussion

### 3.1. Phytochemical Investigation

Phytochemical investigation suggested the presence of different types of primary phytoconstituents in the mixture like flavonoids, terpenoids, phenol, tannins, and reducing sugar. The generalized tested protocols cited in the text were used for superficial screening of SL extract. The phytochemical investigation suggested the presence of impurities and adulterants within an extract. In the present attempt, the phytochemical analysis is presented in Table 1, which shows supplied SL extract was pure and did not contain any impurities. The phytochemical investigation suggested the presence or absence of primary or secondary metabolites having prominent pharmacological activity. The chemical composition of SL was established by phytochemical investigation. The preliminary phytochemical investigation was in accordance with reported literature [26].

### 3.2. Fourier Transform Infrared Spectroscopy (FTIR)

**Figure 1:**
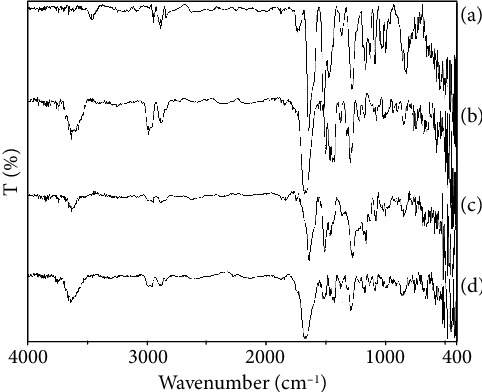
(a) FTIR spectra of silymarin, (b) PVP K30, (c) physical mixture, and (d) SSD3.

An FTIR spectrum was helpful for preliminary identification of SL and comparative evaluation of vibrational frequencies. The stretching and bending vibrational frequencies emerge from SL was depicted in Figure 1(a). Intermolecular interaction in physical mixture between solids was analyzed by vibrational changes during FTIR analysis. The frequencies can be served as probe for identification of surface functional groups and their interactions [29]. The broad intense peak that appeared at 3457 cm^−1^ was specifically due to oxygen-containing functionality of SL. The broad peak represented –OH stretching vibration mode. The distinct peaks observed at 2942 cm^−1^ and 2880 cm^−1^ were due to the presence of CH-CH stretching vibration. The reactive flavonolignon ketone showed strong intense peak at 1639 cm^−1^. A small intense peak at 1365 cm^−1^ and 1278 cm^−1^ was due to –OH bending and C-O-C stretching, respectively. Two conjugated peaks emerged at 1509 cm^−1^ and 1467 cm^−1^ representing aromatic ring stretching vibrations [21]. An intense peak appeared at 1731 cm^−1^ showed the presence of C-O stretch from aromatic ring structure. The in-plane vibration stretching of –C-H was observed at 1085 cm^−1^ corresponding to flavonolignans, while the peaks that appear at 996 cm^−1^ were due to benzopyran ring [30]. The SL primarily reveals the presence of polyphenolic moiety and confirms its structural vibrational frequencies from FTIR spectra [4].

FTIR spectra of PVP K30 are depicted in Figure 1(b). A broad spectrum at 3457 cm^−1^ and 1639 cm^−1^ was observed due to the presence of –OH stretching and carbonyl stretching vibrations, respectively, while two conjugative peaks at 2977 cm^−1^ and 2878 cm^−1^ are designated for CH-CH stretching from polymeric chain. A sharp peak at 1670 cm^−1^ was observed due to C=O stretching vibration mode [31]. Three conjugative sharp peaks around1470–1437 cm^−1^ were due to the presence of C-H bending. A strong band at 1290 cm^−1^ was observed due to the in-plane OH bending vibration and interestingly involved in the interaction. The interaction may drop the crystallinity of phytoconstituent [32]. A small band at 1072 cm^−1^ emerges due to C-O stretching vibration. All the reported vibrational frequency confirmed the structural conformity of PVP K30 [33]. Physical interaction of SL and PVP K30 was analyzed using FTIR spectroscopy, and spectral elucidation is represented in Figure 1(c). Characteristic vibrational frequencies of both components were found to be superimposed in physical mixture that indicated no interaction between SL and PVP K30. Intensity of peaks increased or decreased simultaneously based on overlapping structure available. The physical mixture did not show any types of interaction between SL and PVP K30 [31]. The overlapping hydroxyl stretch was observed at 3624 cm^−1^ with decreasing CH-CH stretching at 2945 cm^−1^ and 2874 cm^−1^. The C-O stretching at 1638 cm^−1^ was elongated, and a sharp peak was observed. A prominent peak was shown at 1274 cm^−1^ that represents C-N stretching. The decrease in peak intensity may be due to overlapping bands having similar functional groups [21]. FTIR spectrum of SSD3 is represented in Figure 1(d), which displayed distinct vibration intensity describing the successful formation of solid dispersion. The spectral intensity slightly increases, which may possibly be due to the weak Van der Waals interaction or hydrogen bonding between water molecule and PVP K30-encapsulating SL [31, 34]. A broad peak appeared at 3628 cm^−1^, which showed strong interaction between SL and PVP K30 representing hydroxyl stretching. Two strong peaks observed at 2952 cm^−1^ and 2880 cm^−1^ showed CH_2_-CH_2_ stretching vibration; intensity slightly decreased after interaction. A strong peak observed at 1670 cm^−1^ was due to C=O stretching, which may be emerged from both SL and PVP K30. The small peak was observed at 1508 cm^−1^ represents N binding due to the presence of C-N in the structure of PVP K30 [34]. Two new peaks formed at 1423 cm^−1^ and 1374 cm^−1^ represent C-H bending were emerged due to physical interaction. A strong peak at 1290 cm^−1^ was emerged due to C-O bending vibration preserved from the PVP K30 [21, 31].

### 3.3. X-Ray Diffraction

**Figure 2:**
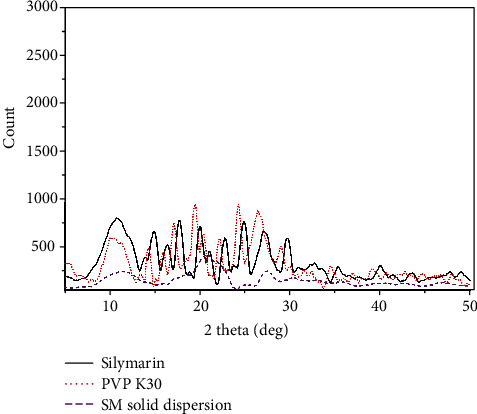
X-ray spectra of pure silymarin, PVP K30, and silymarin solid dispersion.

Powdered XRD distinctively analyzes crystalline and amorphous transition in pharmaceutical products and processes. The X-ray diffraction spectrum of pure SL is depicted in Figure 2. Strong crystalline intense peaks were observed about 10.67°, 14.95°, 16.33°, 17.71°, 20.09°, 22.85°, 24.93°, 27.18°, and 29.73° at 2*θ* angle [21]. The intense peaks represented the complete crystalline structure of SL at following Miller indices, etc. Similarly, PVP K30 was amorphous in nature, but due to the hygroscopic nature of PVP, few intense peaks were observed during analysis. The slight crystallinity was observed at 10.32°, 14.35°, 15.95°, 17.2°, 19.53°, 22.25°, 24.28°, 26.48°, and 28.95° at 2*θ* angle. The intense peaks assigned for respective miller indices confirm the crystalline nature [34]. The resultant solid dispersion formed by solvent evaporation methods can be able to successfully convert into amorphous form. The increased diffraction width in the diffraction angle suggested the decrease in crystallite size. In Figure 2 representing SSD, the crystalline bands at 11.25°, 20°, 27.43°, and 32.83° have broadened area which suggested that the small silymarin crystallites were encapsulated inside the polymeric structure [35]. In terms of solubility characteristics, the decrease in peak intensity or broadening of peak was preferably observed in amorphous forms and responsible for enhancement of solubility [21].

### 3.4. Solubility and Percentage Drug Content

**Figure 3:**
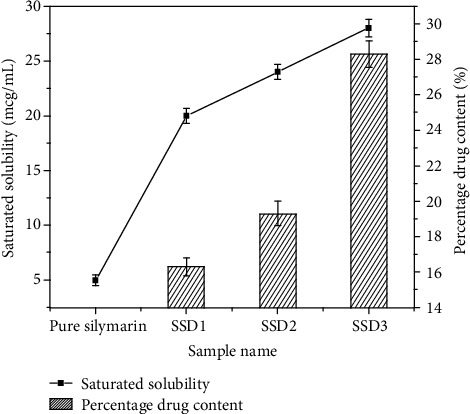
Saturated solubility and percentage drug content of pure silymarin and prepared SSD1, SSD2, and SSD3.

Saturated solubility analysis of pure SL and prepared SSD is depicted in Figure 3. Aqueous solubility of SL was determined by shake flask method and observed to be 5 ± 0.5 *μ*g/mL. The solubility of SL in aqueous media was found concentration dependent. Increase in concentration of PVP K30 leads to increase in solubility of SL. The SSD3 having a concentration ratio of 1 : 3 showed more than 5-fold increase in solubility, while SSD1 and SSD2 had lower concentration that varied within 20–24 *μ*g/mL, respectively. The solubility study described that SSD successfully enhanced in the presence of PVP K30 [21]. Figure 3 also represents percent of silymarin available in SSD1, SSD2, and SSD3. The percentage of drug was determined by UV-Vis spectrophotometric study and used for drug release as well as pharmacological evaluation. The percentage of SL loaded in solid dispersions was found to be 16.31, 19.3, and 28.3% with different batches, SSD1, SSD2, and SSD 3, respectively. SL loading efficiency was increased with increasing concentration of PVP K30 [35].

### 3.5. *In Vitro* Drug Release

**Figure 4:**
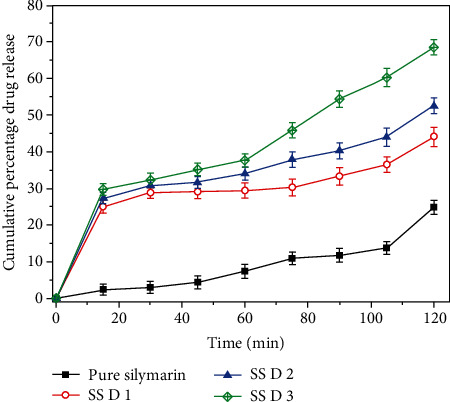
*In vitro* drug release study of pure silymarin in comparison with prepared SSD1, SSD2, and SSD3.

Comparative drug release of pure SL and solid dispersion is depicted in Figure 4. Pure SL was released up to 24.86% within 2 h, while the solid dispersion with variable ratios released the maximum amount of SL into the dissolution medium. At 1 : 1 ratio (SSD1) release 44.1% of SL within 2 h enhanced the dissolution rate by 1.77-folds compared to pure SL. The interaction between PVP K30 and SL was stronger that led to increase in the saturation solubility almost by 5-folds as depicted in Figure 4. This helped to evaluate the dissolution rate of prepared SSD with increasing dissolution in the presence of PVP K30. The release of SL from the inner cores of PVP K30 was specifically by erosion as up to 25% of SL was released instantly within the first 15 min. Furthermore, increasing PVP K30 increased the dissolution and release of SL into dissolution media as verified from Figure 4. SSD3 showed enhanced dissolution rate up to 2.8-fold increment compared to pure SL. SSD3 released more than 83% of SL within 2 h [36].

### 3.6. *In Vivo* Pharmacological Activity

#### 3.6.1. Anti-Inflammatory Activity

The various stimuli like trauma, immunogenic reactions, and infection of microorganism initiate the inflammatory response as indication of physiological response among the individuals [25]. Fundamentally, the inflammatory response is characterized by enzymatic stimulation of arachidonic acid pathway and synthesis and release of eicosanoids like prostaglandins, thromboxanes, and leukotrienes by cyclooxygenase and 5-lipoxygenase enzymes [37]. Moreover, the drugs with central antipyretic and anti-inflammatory action cause downregulation of fever and inflammation, although the role of the antioxidant mechanism pathway in mediating the action of such agents has not yet been elucidated. Considerably, there are two phases of inflammation like early and delayed. The phase is associated with the synthesis of histamine, 5-hydroxytryptamin, bradykinin, and cyclooxygenase, whereas delayed phase is associated with infiltration of neutrophils and continuous production of metabolites of arachidonic acid [39].

**Figure 5:**
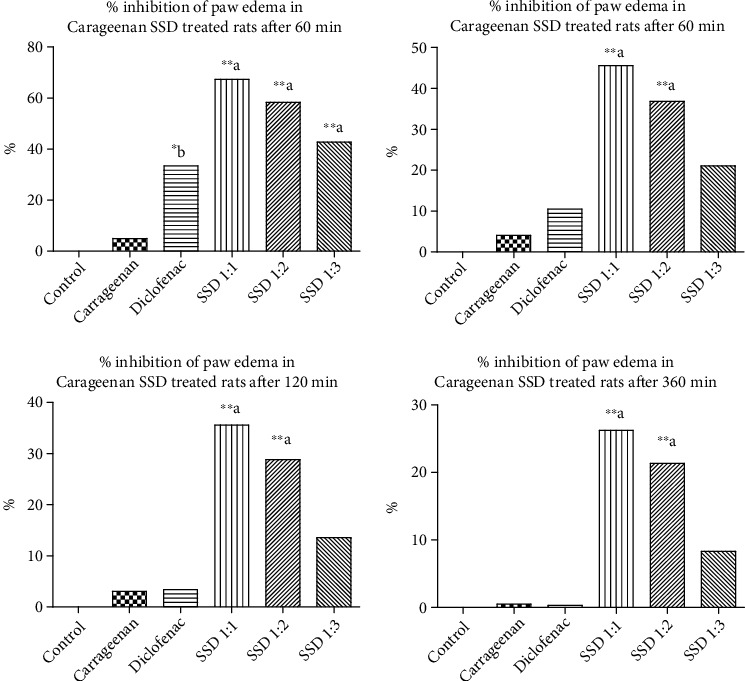
*In vivo* anti-inflammatory activity of silymarin solid dispersion. Carrageenan-induced animal model shows response measurement time points at 30 min, 60 min, 120 min, and 360 min, respectively. Statistical control: ^∗b^*p* < 0.05 compared with control; ^∗∗a^*p* < 0.001 compared with control.

*(1) Carrageenan-Induced Rat Paw Edema*.  Figure 5 and Table 2 show carrageenan-induced rat paw edema anti-inflammatory response of prepared SSD in rats at variable interval of time. The SSD showed promising inhibition activity against carrageenan-induced rat paw edema. The solubility and dissolution were enhanced in the presence of PVP K30, which also provided permeability characteristics through the skin surface. After preparation of SSD in variable concentration ratios between SL and PVP K30, it significantly inhibited the edema produced by carrageenan. The wide variety of chemicals is successfully screened with the help of carrageenan-induced rat paw edema for their potential anti-inflammatory effects. The carrageenan-induced rat paw edema develops the biphasic response of inflammation [40]. The initial phase last for about 1 h and is thought to be mediated through release of various chemical mediators like histamine and serotonin. On the other hand, the later phase is brought about by the release of substances like prostaglandins. Based on this, the second phase may be explained by an inhibition of cyclooxygenase or development of antioxidative properties [38, 39].

From Figure 5, statistical significance in comparison to standard SL, SSD has higher percentage. The different time points recorded during experiment such as 30, 60, 120, and 360 min provide longer time effects. The positive control diclofenac showed the highest inhibition rate up to 0.33% after 360 min, while SSD (1 : 3) inhibited up to 8.33%. Initially, after 30 min of administration of SSD, % inhibition of edema was decreased from 67.31% (SSD1), 58.36% (SSD2), to 42.80% (SSD3) with respect to concentration of PVP K30 increased. At 360 min, the percent inhibition lowered to the values of 26.22% (SSD1), 21.31% (SSD2), and 8.33% (SSD3), respectively.

#### 3.6.2. *In Vivo* Hepatoprotective Activity

*(1) Carbon Tetrachloride Model*. Despite its mild analgesic and antipyretic effect [40, 41], the oxymetabolite of carbon tetrachloride (CCl_4_) has potential to cause liver toxicity through the depletion of GSH-Px level. Also, these metabolites cause lipid peroxidation and induce death of liver cells resulting in an elevation of serum enzyme AST, ALT, and ALP [24]. CCl_4_ starts peroxidation in adipose tissue that results in loss of integrity of lipid membrane and starts damaging hepatic tissue. The sequential liver damage was reflected by reduction in protein synthesis, metabolic enzyme inactivation, etc. Hepatocellular injury was measured by quantifying levels of bilirubin and other proteins and enzymes by biochemical test [42, 43], and the values are displayed in Table 3.

**Figure 6:**
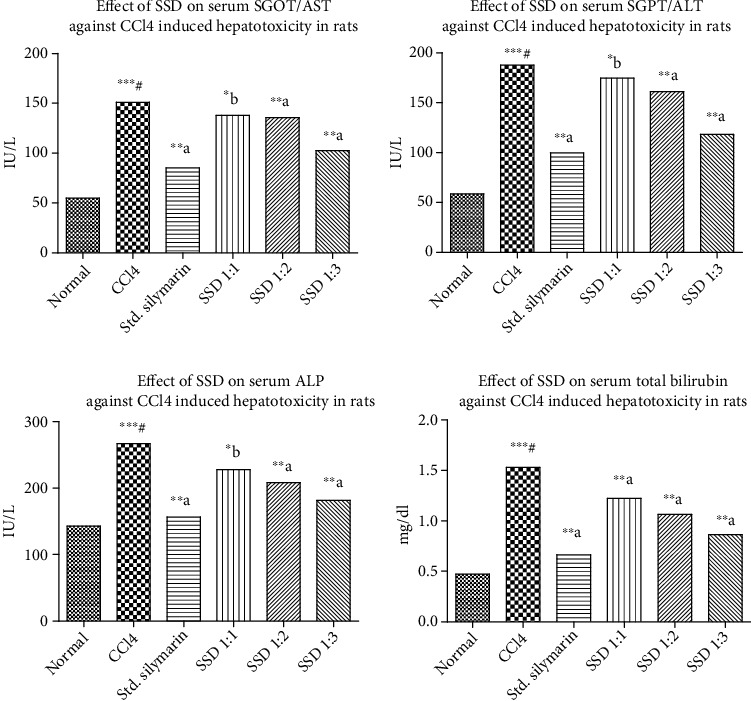
Carbon tetrachloride- (CCL_4_-) induced hepatotoxicity assessment on silymarin and prepared SSD: effect on SGOT (AST), effect on SGPT (ALT), effect on ALP, and effect on total bilirubin against CCL_4_. Statistical control: ^∗∗∗#^*p* < 0.0001 compared with control by the Student unpaired “*t*”-test; ^∗b^*p* < 0.05 compared with CCL_4_; ^∗∗a^*p* < 0.001 compared with CCL_4_.

The mechanism of SL acts by maintaining the integrity of hepatocellular membrane and prevents permeation of toxins into the interior section of the liver to avoid further cellular damage. SL enhances ribosomal protein synthesis by activating nucleolar polymerase A (NPA). The NPA helps to start synthesis of hepatocytes and regenerating potential of the liver [44]. SSD has significantly reduced the levels of serum SGOT/AST, SGPT/ALT, ALP, and total bilirubin as represented in Figure 6. The enhanced solubility in the presence of PVP K30 at variable concentration significantly increased the bioavailability and permeability characteristics compared to pure SL. The CCL_4_ administration elevated the level of these biomarkers and started reducing efficiency of the liver. The SSD provided a stronger therapeutic potential by reducing the inflammatory response by preventing penetration of toxins like trichloromethyl and trichloromethylperoxy. Comparatively, SSD (1 : 3) showed maximum reduction in the level of serum biomarkers. Protective ability of silymarin was enhanced after encapsulating with PVP K30.

**Figure 7:**
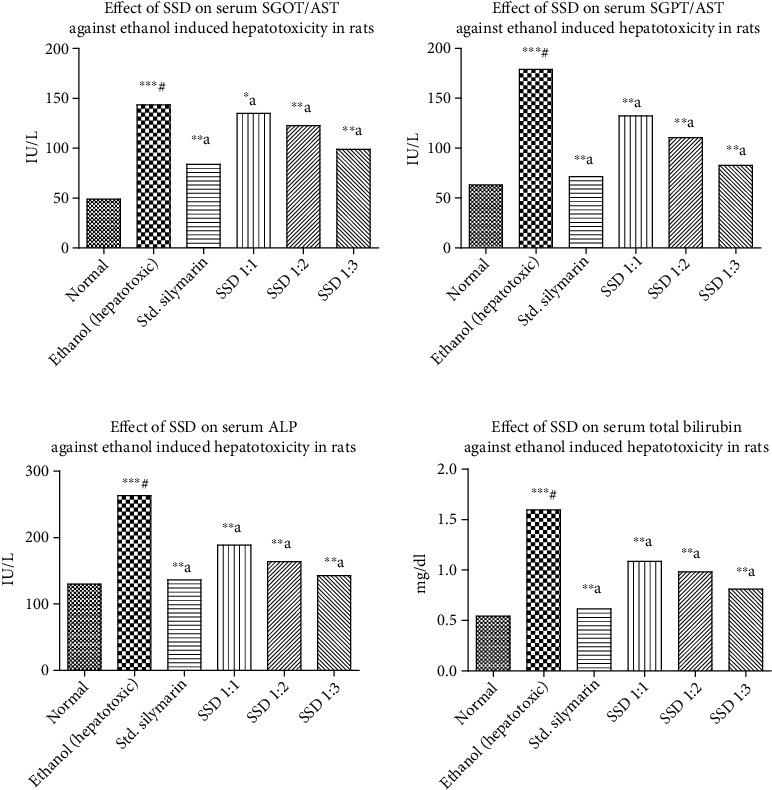
Ethanol-induced hepatotoxicity assessment of silymarin and prepared SSD: effect on SGOT/AST, effect on SGPT/ALT, effect on ALP, and effect on total bilirubin concentration, respectively. Statistical control: ^∗∗∗#^*p* < 0.0001 compared with control by the Student unpaired “*t*”-test; ^∗∗b^*p* < 0.05 compared with ethanol; ^∗∗a^*p* < 0.001 compared with ethanol.

*(2) Ethanol-Induced Hepatotoxicity in Rats*. Generally, the liver plays a vital role in carrying out enzyme-mediated different metabolic reactions in the body. According to extensive literature, it was observed that ingestion of ethanol causes damage of hepatic cells. The possible underlying mechanism involves the elevation of serum AST, ALT, ALP, and total bilirubin in rats and further structural and functional modulation of liver cells [45, 46]. Ethanol-based hepatotoxicity models were continuously evaluated due to multiple toxic reports available frequently in hospitals. The number of patients is increasing with liver toxicity due to higher consumption of alcohol. Phytomedicines are more effective in the management of liver toxicities. The recovery rate is higher comparative to chemotherapeutic agents. The effects of pretreatment with three SSD in the ratio of 1 : 1, 1 : 2, and 1 : 3 on the ethanol-induced elevation of serum AST, ALT, ALP, and total bilirubin are depicted in Figure 7. There was proportionate reduction in the elevated levels of serum AST, ALT, ALP, and total bilirubin with respect to PVP K30 concentration in the formulations. The SSD (1 : 3) showed higher reduction response due to the enhanced solubility of SL. The biomarker level proportionally decreased as solubility increased with composition ratio ranging from SSD 1 : 1 to 1 : 3. SSD with PVP in the ratio of 1 : 1 (*p* < 0.05), 1 : 2 (*p* < 0.01), and 1 : 3 (*p* < 0.01) significantly prevented the elevated level of serum AST, ALT, ALP, and total bilirubin. Our studies on the ethanol-induced hepatic damage were in accordance with previous reports [47]. This study demonstrated that pretreatment with three SSD in the ratio of 1 : 1, 1 : 2, and 1 : 3 had significantly reduced levels of serum AST, ALT, ALP, and total bilirubin, which were elevated by ethanol administration as shown in Figure 7 and Table 4.

**Figure 8:**
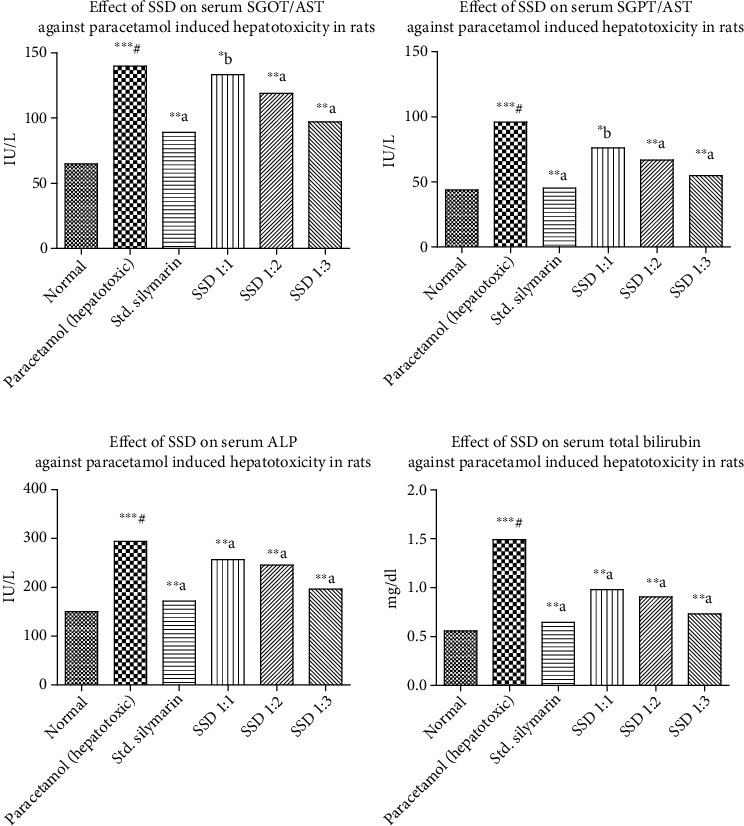
Paracetamol-induced hepatotoxicity assessment of silymarin and prepared SSD: effect on SGOT, effect on SGPT, effect on ALP, and effect on total bilirubin against paracetamol, respectively. Statistical control: ^∗∗∗#^*p* < 0.0001 compared with control by the Student unpaired “*t*”-test; ^∗b^*p* < 0.05 compared with paracetamol; ^∗∗a^*p* < 0.001 compared with paracetamol.

*(3) Paracetamol-Induced Hepatotoxicity in Rats*. Paracetamol is one of the most important hepatotoxic agents reported in the treatment of pediatric patients. Many adverse events and dose dumping complications in the treatment of paracetamol were reported specifically related to toxicity. The biomarkers like SGPT, SGOT, ALT, and serum bilirubin were identified to assess the toxicity level of paracetamol in patients undergoing treatment. Generally, liver toxicity is induced by ingestion of paracetamol and it is routinely employed in the screening of wide range of chemicals for their hepatoprotective activity in rodents. The increased production of serum enzymes in blood stream was associated with central/submissive necrosis of the liver, which caused severe hepatic injury as shown in Figure 8 and Table 5.

## 4. Conclusion

SL is a natural lipophilic molecule, and it has an aqueous solubility of 5 *μ*g/mL. Due to its low aqueous solubility, it has low oral bioavailability (23-47%), and after oral administration, it leads to poor therapeutic application. An attempt was tried to increase the solubility and bioavailability of silymarin by solid dispersion technique using PVP-K30. PVP K30 inhibited the crystallization of SL and produced amorphous solid eutectic mixture for therapeutic applications. The solubility was increased maximum up to 5-fold after encapsulation inside the PVP K30. A variable ratio pertaining to PVP K30 and SL was intact within the solution phase and forms a homogenous mixture. The stronger interactions were highly dissociated in the presence of dissolution medium and enhanced the release of SL from the inner compartment. The dissolution rate was increased by 2.8-fold compared to pure SL. The wettability characteristics and favourable interaction with the solvent molecules helped to dissociate silymarin instantly from the eutectic complex. In the presence of aqueous environment, thereby erosion of PVP K30 took place and released about 23% SL within the first 15 min. The drug release characteristics were increased with an enhanced bioavailability verified by anti-inflammatory and hepatoprotective activities. The local solubilisation effect was prominent and verified by anti-inflammatory response using carrageenan-induced inflammation in rats. All three hepatoprotective models showed reduction in the biomarker response that shows good bioavailability of silymarin in the presence of PVP K30.

## Figures and Tables

**Table 1:** Phytochemical evaluation of silymarin extract.

Sr. no.	Phytochemical test	Observation	Inference
1	Mayer's test	No precipitate observed	Alkaloid absent
2	Dragendorff's test	Absence of orange color	Alkaloid absent
3	Wagner's test	Absence of reddish-brown precipitate	Alkaloid absent
4	Extract+aluminium chloride solution	Yellow-colored solution	Flavonoids present
5	Extract+Aq. NaOH	Yellow-orange color solution, disappears after addition of HCl	Flavonoid present
6	Extract+Conc. H_2_SO_4_	Orange-colored solution	Flavonoid present
7	Extract+Aq. ferric chloride (10%)	Deep blue-colored solution	Phenol present
8	Extract+ferric chloride (5%)	Green black-colored solution	Tannins presents
9	DW+extract+shake vigorously	Absence of foam layer	Saponins absent
10	Salkowski test	Reddish brown color at the interface	Terpenoids present
11	Extract+Conc. HCl	Yellow-colored precipitate not observed	Quinins absent
12	Extract+2 M NaOH	Blue green color not formed	Anthocyanin absent
13	Ninhydrin test	Purple color not observed in solution	Protein absent
14	Benedict's test	Red precipitate forms	Reducing sugar present
15	Iodine test	Purple color not formed after addition of iodine	Polysaccharide absent
16	Extract+2% HCl	No color change	Anthraquinones absent

^∗∗^Note: the entire phytochemical test conducted as per protocol mentioned in [27, 28] without modification.

**Table 2:** *In vivo* anti-inflammatory activity of SSD in rats. *In vivo* carrageenan-induced paw edema in rats (*n* = 6).

Name of group	Edema value (mL) and % of inhibition			
	30	60	120	360
Control	0.8521 ± 0.006241	0.8754 ± 0.004578	0.8625 ± 0.005924	0.8425 ± 0.004231
Carrageenan treated	0.5120 ± 0.005831^∗∗∗^^#^	0.5740 ± 0.006782^∗∗^^#^	0.5940 ± 0.005099^∗∗^^#^	0.6040 ± 0.002449^∗^^#^
Std. diclofenac sodium (10 mg/kg)	0.6860 ± 0.005099^∗^^b^ (33.46%)	0.6260 ± 0.005099 (10.52%)	0.6100 ± 0.003162 (3.38%)	0.6020 ± 0.003742 (0.33%)
SSD 1 : 1	0.8600 ± 0.07785^∗∗^^a^ (67.31%)	0.8280 ± 0.07297^∗∗^^a^ (45.61%)	0.8060 ± 0.06794^∗∗^^a^ (35.59%)	0.7760 ± 0.06990^∗∗^^a^ (26.22%)
SSD 1 : 2	0.8140 ± 0.01939^∗∗^^a^ (58.36%)	0.7820 ± 0.01881^∗∗^^a^ (36.84%)	0.7600 ± 0.01643^∗∗^^a^ (28.81%)	0.7360 ± 0.01749^∗^^b^ (21.31%)
SSD 1 : 3	0.7340 ± 0.008124^∗∗^^a^ (42.80%)	0.6920 ± 0.007348 (21.05%)	0.6800 ± 0.009129 (13.55%)	0.6520 ± 0.008602^∗∗^ (8.33%)

The data is presented as mean ± SEM. ^∗b^*p* < 0.05 compared with control; ^∗∗a^*p* < 0.001 compared with control.

**Table 3:** *In vivo* hepatoprotective study by CCL_4_ model in various biochemical parameters (*n* = 6).

Name of group	AST (IU/L)	ALT (IU/L)	ALP (IU/L)	Total bilirubin (mg/dL)
Normal group	54.92 ± 4.170	58.62 ± 4.284	142.9 ± 2.466	0.4740 ± 0.04501
CCl_4_ group	151.2 ± 4.910^∗∗∗^^#^	187.8 ± 3.545^∗∗∗^^#^	267.0 ± 20.09^∗∗∗^^#^	1.530 ± 0.03742^∗∗∗^^#^
Silymarin treated	85.40 ± 3.103^∗∗^^a^	99.59 ± 1.263^∗∗^^a^	156.7 ± 4.358^∗∗^^a^	0.6640 ± 0.03816^∗∗^^a^
SSD (1 : 1)	138.1 ± 0.9782^∗^^b^	174.6 ± 1.705^∗^^b^	227.9 ± 4.450^∗^^b^	1.222 ± 0.07915^∗∗^^a^
SSD (1 : 2)	135.7 ± 2.565^∗∗^^a^	161.0 ± 1.669^∗∗^^a^	208.4 ± 2.632^∗∗^^a^	1.064 ± 0.03415^∗∗^^a^
SSD (1 : 3)	102.5 ± 1.176^∗∗^^a^	118.2 ± 4.564^∗∗^^a^	181.5 ± 4.432^∗∗^^a^	0.8620 ± 0.05034^∗∗^^a^

Values are expressed as mean ± SEM. ^∗∗∗#^*p* < 0.0001 compared with control by the Student unpaired “*t*”-test; ^∗b^*p* < 0.05 compared with CCl_4_; ^∗∗a^*p* < 0.001 compared with CCl_4_.

**Table 4:** In *vivo* hepatoprotective study by ethanol model on various biochemical parameters.

Name of group	AST (IU/L)	ALT (IU/L)	ALP (IU/L)	Total bilirubin (mg/dL)
Normal	49.04 ± 1.872	63.02 ± 1.286	130.0 ± 1.450	0.5420 ± 0.009165
Ethanol treated	143.5 ± 2.665^∗∗∗^^#^	179.1 ± 2.207^∗∗∗^^#^	262.8 ± 4.046^∗∗∗^^#^	1.596 ± 0.02638^∗∗∗^^#^
Silymarin-treated group	83.90 ± 1.672^∗∗^^a^	71.34 ± 2.970^∗∗^^a^	136.5 ± 2.970^∗∗^^a^	0.6140 ± 0.02786^∗∗^^a^
SSD (1 : 1)	134.9 ± 1.434^∗^^b^	132.3 ± 6.644^∗∗^^a^	188.6 ± 6.259^∗∗^^a^	1.086 ± 0.05750^∗∗^^a^
SSD (1 : 2)	122.7 ± 2.338^∗∗^^a^	110.5 ± 2.478^∗∗^^a^	163.9 ± 2.492^∗∗^^a^	0.9820 ± 0.003742^∗∗^^a^
SSD (1 : 3)	98.87 ± 0.9379^∗∗^^a^	82.74 ± 3.654^∗∗^^a^	142.5 ± 7.257^∗∗^^a^	0.8100 ± 0.04037^∗∗^^a^

Values are expressed as mean ± SEM. ^∗∗∗#^*p* < 0.0001 compared with control by the Student unpaired “*t*”-test; ^∗b^*p* < 0.05 compared with ethanol; ^∗∗a^*p* < 0.001 compared with ethanol.

**Table 5:** In vivo hepatoprotective study by paracetamol model on various biochemical parameters.

Name of group	AST (IU/L)	ALT (IU/L)	ALP (IU/L)	Total bilirubin (mg/dL)
Normal	64.90 ± 2.941	43.87 ± 2.496	150.3 ± 5.004	0.5580 ± 0.03441
Paracetamol-treated group	139.7 ± 1.732^∗∗∗^^#^	95.93 ± 8.108^∗∗∗^^#^	294.0 ± 13.55^∗∗∗^^#^	1.490 ± 0.04012^∗∗∗^^#^
Silymarin treated	89.01 ± 1.790^∗∗^^a^	45.27 ± 4.737^∗∗^^a^	171.8 ± 1.743^∗∗^^a^	0.6440 ± 0.03092^∗∗^^a^
SSD (1 : 1)	133.2 ± 1.132^∗∗^^b^	76.01 ± 2.843^∗^^b^	256.7 ± 3.839^∗∗^^a^	0.9800 ± 0.005477^∗∗^^a^
SSD (1 : 2)	119.1 ± 1.870^∗∗^^a^	66.87 ± 3.418^∗∗^^a^	245.7 ± 7.042^∗∗^^a^	0.9060 ± 0.02839^∗∗^^a^
SSD (1 : 3)	97.14 ± 0.8002^∗∗^^a^	54.79 ± 3.859^∗∗^^a^	196.6 ± 1.886^∗∗^^a^	0.7300 ± 0.01517^∗∗^^a^

Values are expressed as mean ± SEM. ^∗∗∗#^*p* < 0.0001 compared with control by the Student unpaired “*t*”-test; ^∗b^*p* < 0.05 compared with paracetamol; ^∗∗a^*p* < 0.001 compared with paracetamol.

## Data Availability

All the data used to support the findings of this study are included within the article.
